# Case report: Novel compound heterozygosity for pathogenic variants in *MED23* in a syndromic patient with postnatal microcephaly

**DOI:** 10.3389/fneur.2023.1090082

**Published:** 2023-02-07

**Authors:** Emanuela Salzano, Marcello Niceta, Simone Pizzi, Francesca Clementina Radio, Martina Busè, Francesca Mercadante, Sabina Barresi, Arturo Ferrara, Cecilia Mancini, Marco Tartaglia, Maria Piccione

**Affiliations:** ^1^Medical Genetics Unit, AOOR Villa Sofia-Cervello Hospitals, Palermo, Italy; ^2^Genetics and Rare Diseases Research Division, Ospedale Pediatrico Bambino Gesù, IRCCS, Rome, Italy; ^3^Department of Health Promotion, Mother and Child Care, Internal Medicine and Medical Specialties, University of Palermo, Palermo, Italy

**Keywords:** MED23, post-natal microcephaly, epilepsy, whole exome sequencing, case report

## Abstract

Biallelic loss-of-function variants in *MED23* cause a recessive syndromic intellectual disability condition with or without epilepsy (MRT18). Due to the small number of reported individuals, the clinical phenotype of the disorder has not been fully delineated yet, and the spectrum and frequency of neurologic features have not been fully characterized. Here, we report a 5-year-old girl with compound heterozygous for two additional *MED23* variants. Besides global developmental delay, axial hypotonia and peripheral increased muscular tone, absent speech, and generalized tonic seizures, which fit well MRT18, the occurrence of postnatal progressive microcephaly has been here documented. A retrospective assessment of the previously reported clinical data for these subjects confirms the occurrence of postnatal progressive microcephaly as a previously unappreciated feature of the phenotype of *MED23*-related disorder.

## Introduction

The mediator complex subunit 23 gene (*MED23*; MIM# 605042) encodes a protein functioning as a tail module mediator complex, a multisubunit co-activator that is implicated in many cellular processes as a part of the core transcription machinery (1, 2). Functional inactivation of any of the different subunits of the mediator complex results in the dysregulation of genes controlling the developmental processes, including early brain development and neuroplasticity (3, 4). Structurally, MED23 is composed of 25 HEAT repeat-like motifs organized into 4 α-solenoids generating a large MED23 core region (3-HEAT, 5-HEAT, 6-HEAT, and C-HEAT) and an N-terminal domain (N-HEAT) protruding from the core region (5). To date, 11 individuals and < 10 pathogenic variants in *MED23*, mostly missense, have been reported thus far (3, 6, 7, 9, 10) (Table 1); https://www.hgmd.cf.ac.uk/ac/gene.php). In most cases, the pathogenic substitutions affect the residues clustering in N-HEAT, 3-HEAT, and 5-HEAT and are believed to exert the loss-of-function (LoF) of MED23 function (3). Due to a small number of the reported individuals with biallelic *MED23* variants, the clinical phenotype of the disorder is not fully delineated yet. Since the first reports that correlated biallelic *MED23* mutations to a non-syndromic autosomal recessive neurodevelopment disorder (3), descriptions of other cases have emerged the hypothesis of a more complex phenotype depending on the type and position, and effect of *MED23* variants (6, 7, 9, 10). Thus, while microcephaly, axial hypotonia, spasticity, choreoathetosis, dystonia, and epilepsy are described as the features of the “classical phenotype,” other features such as developmental delay (DD) and intellectual disability (ID), screaming spells, electroencephalography (EEG) abnormalities, and epilepsy have commonly been documented in affected subjects (6, 7, 9). Notably, speech delay has also been sporadically reported in some instances (6, 7, 10).

**Table 1:** Summary of subjects and our case with *MED23*-associated disorder.

**Clinical report**	**Hashimoto et al**. **(****3****)** **family** [4 Female and 1 Male^*^]		**Trehan et al. (6)**	**Trehan et al. (6)**	**Lionel et al. (7)**	Riazuddin et al. (8) PKMR85 Family^**^	**Demos et al. (9) 069 P**	**Hashemi-Gorji et al. (10)**	**Current report**
Sex	F	F	M	M	M	3M and 1F	M	M	F
Consanguinity	Yes	Yes	No	No	Yes	Yes	No	Yes	No
*MED23* variant (NM_004830.3)	c.1832G > A; p.R611Q / c.1832G > A; p.R611Q	c.1832G > A; p.R611Q / c.1832G > A; p.R611Q	c.3638A > G; p.H1213R / c.3988C > T; p.R1330^*^	c.3638A > G; p.H1213R / c.3988C > T; p.R1330^*^	c.1919A > G; p.Q640R / c.1919A > G; p.Q640R	c.506A > G; p. Y169C / c.506A > G p.Y169C	c.382G > A; p.G128R / c.539C > A; p.A180D	c.670C > G; p.R224G / c.670C > G; p.R224G	c.1831C > T; p.R611W / c.383G > A; p.G128E
Age of onset	NR	NR	Early infancy (12 months)	Infancy (22 months)	5 months	NR	51.5 months	Infancy	Birth
Age at diagnosis	39 years	41 years	11 years	5 years	7.5 years	NR	NR	24 years	6 years
OFC at birth	NR	NR	50th centile	10th centile	NR	NR	NR	NR	50thcentile
OFC at diagnosis	Normal	Normal	75th centile	5th centile	< 2nd centile	NR	NR	< 3rd centile	< 3rd centile
Clinical features	GDD mild to moderate ID (unable to write and read and to look after their financial affairs)	GDD mild to moderate ID (unable to write and read and to look after their financial affairs)	Profound ID, spasticity, axial hypotonia, dystonia, speech delay, ventricular septal defect	Profound ID, axial hypotonia, spasticity, choreoathetosis, speech delay, CVI, atrial septal defect	GDD, microcephaly, axial hypotonia, spasticity, no independent walking, absent speech	ID	GDD, profound ID, spastic CP, dystonia, CVI, microcephaly, G-tube fed	GDD, profound ID hypotonia in hands, microcephaly, speech delay, spasticity	GDD, profound ID secondary progressive microcephaly, axial hypotonia, spasticity, no independent walking, absent speech
Seizures/EEG features/treatment	None/normal/none	None/normal/none	Screaming spells/unprovoked and photic-provoked epileptiform abnormalities/none	Screaming spells/disorganized slow background, bilateral frontal epileptiform abnormalities/none	Generalized tonic seizures/spike and sharp-wave complexes over the bilateral parasagittal chain/refractory epilepsy treated with ketogenic diet (age of onset: 8 months)	None	Generalized tonic, tonic-clonic/drug therapy responsive (age of onset: 51.5 months)	None/NP	Generalized tonic seizures/global slow and hypo-structured activity/poorly drug therapy responsive (age of onset: 6 months)
Brain MRI	Normal	Normal	Pontine hypoplasia	Pontine hypoplasia, thin corpus callosum, temporal lobe hypomyelination	Thin corpus callosum, delayed myelination	NR	Normal	NP	Delayed myelination

CP, cerebral palsy; CVI, cortical visual impairment; ID, intellectual disability; GDD, global developmental delay; NR, not reported; NP, not performed; OFC, occipital-frontal head circumference.

^*^Hashimoto et al. extensively studied two sisters among the five affected individuals in the family.

^**^Riazuddin et al. reported the same clinical feature for all the affected individuals in the family.

In this study, we report an additional case with novel biallelic variants in *MED23* and review the previously reported series of patients, providing a more accurate delineation of the clinical portrait of the *MED23*-related disorder and suggesting a new diagnostic sign.

## Clinical report

**Figure 1:**
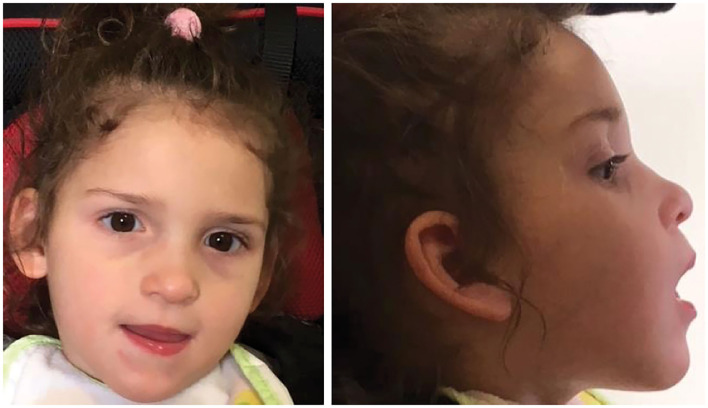
Cranio-facial phenotype of the case including up-slanting palpebral fissures, epicanthal fold, depressed nasal bridge anteverted nares, long philtrum, low-set and posteriorly rotated ears.

The patient is a 5-year-old girl, the second child of healthy, non-consanguineous parents (Figure 1). She was born at 38 + 2 weeks of gestation by cesarean delivery. Apgar scores were 9 and 10 at 1st and 5th min, respectively. Her birth weight was 2,900 g (25th−50th percentile), length was 47 cm (10th−25th percentile), and head circumference was 34 cm (50th percentile). At birth, she showed axial hypotonia and presented with an increased peripheral tone by the age of 2 months.

Despite the normal growth parameters at birth, from the first months of life, a progressive reduction in the growth of head circumference was noted (3rd percentile, at 10 months, reaching the four standard deviations (SDs) below the average at the age of 5 years).

Regarding her psychomotor profile, from early infancy, she exhibited delayed development globally. To date, she has not acquired speech development and independent walking.

She started suffering at the age of 6 months with generalized tonic seizures characterized by loss of contact with environment, right head deviation, and eye turning, followed by post-critical vomiting. She undertook a therapy with valproic acid and clobazam, having only partial control of seizures. EEG showed interictal spikes and sharp waves over the right central posterior and occipito-temporal regions mixed to a global slow and hypo-structured activity with a tendency to the contralateral generalization. Evoked visual potential and auditory brainstem response studies were normal. Brain magnetic resonance imaging (MRI) (performed at seizure onset) showed delayed myelination in the absence of any obvious brain malformation. Heart and abdominal ultrasounds were normal. Biochemical investigations, including ammonium, lactate, plasma amino acids, acylcarnitine profile, urine organic acids, cholesterol metabolism, and congenital disorders of glycosylation, were normal.

She was unsuccessfully studied by the array-CGH and methylation analysis for Angelman syndrome as first-tier tests, followed by the single *UBE3A* and *MECP2* genes' analysis. She was also investigated by a custom-NGS-panel for epilepsy as part of the diagnostic work-up, which was uninformative. Based on the complex and unclassified phenotype and negative findings, she was enrolled in the Undiagnosed Patients Program at the Ospedale Pediatrico Bambino Gesù, Rome, Italy.

## Materials and methods

### Clinical data and ethical compliance

Informed consent for genetic analysis, clinical data, and images was obtained from the family, and it is secured at the Villa-Sofia-Cervello Hospitals (Palermo, Italy). Clinical investigations were conducted according to the Declaration of Helsinki. Genetic analyses were performed in the context of the “Undiagnosed Patients Program,” an initiative directed to accelerate the diagnosis of patients with unsolved clinical conditions, under approval by the Institutional Ethical Committee of the Ospedale Pediatrico Bambino Gesù (1702_OPBG_2018), Rome. Physical assessment was performed by experienced clinical geneticists. Clinical data, pictures, DNA specimens, and other biological materials were collected, used, and stored after the signed informed consent from the participating subjects/families were obtained.

### Genomic analysis

Genomic DNA from the leukocytes of the patient and both parents was extracted using MagPurix Blood DNA Extraction Kit 1200 (Resnova), following the manufacturer's protocols. Targeted enrichment was carried out using SureSelect ClinicalExome V.2 (Agilent), and parallel sequencing was performed on an Illumina NextSeq550 platform. The trio-based whole exome sequencing (WES) data analysis was performed using an *in-house* implemented pipeline, which mainly takes advantage of the Genome Analysis Toolkit (GATK V.3.7) (11) framework, as previously reported (12–14). The high-quality variants were filtered against the public databases (dbSNP150 and gnomAD V.2.0) to retain private and clinically associated variants, annotated variants with unknown frequency or having MAF < 0.1%, and occurring with a frequency < 2% in an *in-house* database including frequency data from ~2,500 population-matched WES. SnpEff toolbox (V.4.3) (15) was used to predict the functional impact of variants, which were filtered to retain only those located in the exons with any effect on the coding sequence, and splice site regions (variants located from −3 to +8 with respect to an exon-intron junction). Functional annotation of variants was performed using SnpEff and dbNSFP (V.3.5) (15–17). The functional impact of variants was analyzed by the Combined Annotation Dependent Depletion (CADD) V.1.3, M-CAP V.1.0, Revel, and InterVar V.2.0 algorithms (18–20). Genes carrying rare/private variants predicted to be deleterious by CADD or M-CAP and Revel and Metadome algorithms were prioritized according to the family history and possible inheritance models (20), following the most recent guidelines by the American College of Medical Genetics and Genomics (ACMG) (21). WES statistics are reported in Supplementary Table S1. Variant validation and segregation analysis were performed by Sanger sequencing.

## Results

### Exome sequencing

**Figure 2:**
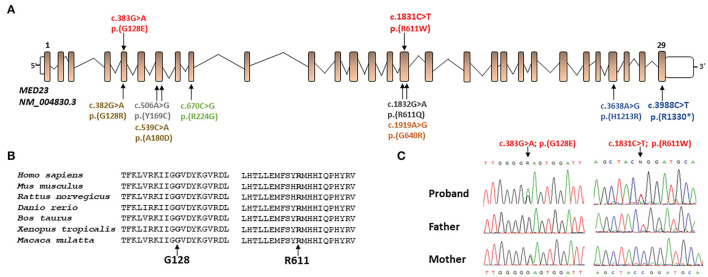
**(A)** Schematic representation of the *MED23* transcript (*NM_004830.3*) and location of all mutations present in literature (in black and gray mutations related to milder phenotypes, in orange, yellow, green, and blue the ones related to more severe phenotype) and novel mutations described in this report (in red). **(B)** Protein sequence alignment of human MED23 with its orthologs and conservation of the affected residues (from the top: NP_004821.2; NP_001159888.1; XP_008756834.1; XP_021324080.1; XP_024852307.1; XP_002936353.2; and XP_014992795.1). **(C)** The *MED23* electropherograms at the genomic DNA level of all family members showing the novel *MED23* mutations identified by WES.

Whole exome sequencing analysis allowed us to identify the compound heterozygosity for two functional relevant variants in *MED23* (NM_004830.3, c.383G > A, p.Gly128Glu; c.1831C > T, p.Arg611Trp) as the event underlying the neurodevelopmental condition. The maternally inherited variant (c.383G > A) localized in exon 5 (Figure 2) has been reported in the public databases (gnomAD v2.1.1, rs762893295) with MAF = 0.000007956 and annotated as uncertain clinical significance (VUS) according to the ACMG recommendations (Table 2). By *in silico* predictions, the change was annotated damaging (CADD = 25.2), affecting the conserved Gly128 within the N-HEAT domain, which implicated protein–protein interactions (5). Similarly, the paternally variant (c.1831C > T) lying in exon 16 (Figure 2) has been reported in a population database (rs761672482) with MAF = 0.00001592 and is considered likely pathogenic (Table 2). The substitution (p.Arg611Trp) was also predicted damaging (CADD = 34), affecting the conserved Arg611 within the 5-HEAT motif, which is required for controlling the orientation and transcriptional activity of the protein (5). Of note, WES analysis also revealed two variants in CFTR (^*^602421): c.489 + 3A > G (rs377729736; CS002122), and c.2991G > C; p. Leu997Phe (rs1800111; CM920171) but were not considered to be related to the developmental and epileptic encephalopathy phenotypes observed in the patient.

**Table 2:** Single-nucleotide variants involving MED23 identified in the present study.

**Genomic coordinates (GRCh37)**	**Variant type**	**Coding variant**	**Exon**	**Amino acid change**	**rsID**	**Max AF**	**CADD^a^ (phred)**	**M-CAP^a^**	**Revel^a^**	**Metadome^b^ d_n_/d_s_**	**ACMG Guidelines^c^**	**MED23 predicted effect**
chr6:131944504C > T	SNV	c.383G > A	5	p.Gly128Glu	rs762893295	0.000008	25.2	0.039	0.53	0.76	VoUS; PP2, PP4, PM2, PM5	Intra-helical hydrogen bond disruption
chr6:131924270G > A	SNV	c.1831C > T	16	p.Arg611Trp	rs761672482	0.000016	34	0.32	0.91	0.25	Likely Pathogenic; PP2, PP3, PP4, PM2, PM5	Inter-chain hydrogen bonds disruption

^a^Variants were predicted to be “deleterious” by Annotation Dependent Depletion (CADD) v.1.3, M-CAP v1.0 and/or Rare Exome Variant Ensemble Learner (REVEL) (23). Scores > 15 (CADDphred) or > 0.5 (REVEL) predict that the sequence change has a significant impact on protein structure and function.

^b^d_N_/d_S_ metric detects evolutionary pressure in protein-coding regions and genomes and is used to measure genetic tolerance (24). Residues with d_N_/d_S_ values < 0.53 are considered to map within regions “intolerant” to nonsynonymous variation.

^c^ACMG classification was assessed using Franklin Genoox (https://franklin.genoox.com/clinical-db/home).

## Discussion

Biallelic LoF variants of *MED23* underlie a neurodevelopment disorder characterized by a clinically wide phenotypic spectrum (6). However, the occurrence of some neurological features remains undefined. To the best of our knowledge, 11 individuals and 9 pathogenic *MED23* variants have been reported thus far (3, 6–10) (Table 1). In the present study, we report an additional subject carrying two compound heterozygous *MED23* variants and displaying a clinical phenotype that only partially overlaps with MRT18 (Table 1). Both changes are rare and predicted to dramatically impair the function of the mature MED23. Indeed, while the p.Gly128Glu change is believed to disrupt the intra-helical hydrogen bonds, destabilizing the 8th helix of the N-HEAT domain, the p.Arg611Trp substitution is predicted to affect the intra-chain interactions within the 5-HEAT motif of the core MED23, possibly impairing its function compromising the activity of the entire mediator complex (5). Of note, other pathogenic MED23 missense changes affecting both Gly128 and Arg611 residues (p.Gly128Arg and p.Arg611Gln, respectively) have been reported in subjects with MRT18 (3, 9) (Table 1). It is intriguing as the most frequent pathogenic *MED23* variants cluster on exons 5, 7, 16, and 29 (Figure 2) and affect specific residues of the protein, suggesting possibly the presence of mutation hotspots in the gene. A more robust and unbiased study on the distribution of *MED23* mutations could confirm this observation.

The clinical phenotype of the *MED23*-related disorder is overall complex, and a subgroup of features is likely to be inconsistent with what is expected in MRT18. Indeed, besides the “classical phenotype” (i.e., microcephaly, axial hypotonia, spasticity, choreoathetosis, dystonia, and epilepsy), a number of additional signs including severe ID, speech delay, and brain anatomic defects (i.e., delayed myelination and thin corpus callosum) have sporadically been documented and emerged to be possibly the result of a pleiotropic effect of defective MED23 (6, 22). There is a growing interest in delineating the clinical spectrum of the *MED23*-related disorder and defining the genotype–phenotype correlations (6, 10, 22). By a review of the clinical features of the previously reported cases (11 subjects), we provide data of features that more frequently may occur in this neurodevelopmental disorder (Table 1). Half of the molecularly-confirmed MRT18 individuals (6/11) together with the present case show the defective functioning of the neurological system including profound ID, early onset DD with axial hypotonia followed by an increased peripheral tone (spasticity) sometimes associated also with motor defects (purposeless on-rhythmic movements and/or dystonia), and visual impairment. Of note, speech impairments may also variably occur (5/11). Furthermore, out of seven individuals (including our affected subject), six presented EEG-epileptiform abnormalities indicating moderate-to-severe cerebral dysfunction with a disorganized slow background and predisposition to partial or generalized seizures. The age of onset of EEG abnormalities is variable, ranging between 8 months and 11 years. Of note, consistent with other previous reports, our patient showed early onset seizures (6 months), suggesting the possibility of a double clustered age of onset (6 months and 4–5 years of age). Studies with a larger cohort however are required to verify this hypothesis. In addition, drug-resistant epilepsy (DRE) has been documented in 2/3 cases, even though a long-term seizure freedom has been reached with the ketogenic diet in one of them (7). Notably, epilepsy is more often documented in individuals with brain anatomical defects such as pontine hypoplasia, abnormal myelination, and thin corpus callosum (4/5 affected individuals), indicating that epilepsy may be secondary to diverse anatomic brain defects. Consistent with the present case, craniofacial dysmorphism seems to be infrequent in the disorder (Figure 1). Microcephaly has been documented in a small number of affected subjects (4/11), even though data on the occipital-frontal head circumference (OFC) were not available for all subjects. Our patient had a normal head size at birth, but stagnation in head growth resulted in acquired microcephaly. At the last evaluation, her head circumference was reduced up to four standard deviations (SDs) below the average at the age of 5 years. Postnatal microcephaly is a typical feature of some neurodevelopmental disorders (e.g., Angelman syndrome, *MECP2-, CASK-, CDKL5-, FOXG1-, SLC9A6-*, and *TCF4*-related disorders). Progressive microcephaly had never been reported in subjects with *MED23* mutations (Table 1), and thus, we speculate on the possibility of the occurrence of this feature in the disorders. This may be not surprising as defective MED23 is known to impair the abilities of mediator complex to interact with enhancer-bound transcription factors, such as TCF4 and ELK1, resulting in the dysregulation of expression of a subset of fine-tuned immediate-early genes (e.g., *JUN* and *FOS*), which are required for brain development and growth (3). More data, however, are required to confirm the postnatal microcephaly as a key feature of *MED23*-related disorder.

In conclusion, the clinical assessment of an additional subject with biallelic *MED23* variants allows us to expand the phenotype of the disorder, thus contributing to define better its neurodevelopmental impairment. In addition, our data suggest the occurrence postnatal microcephaly that may possibly be considered in the *MED23*-related disorders.

## Data availability statement

The datasets presented in this article are not readily available because of ethical and privacy restrictions. Requests to access the datasets should be directed to the corresponding author.

## Ethics statement

The studies involving human participants were reviewed and approved by Institutional Ethical Committee of the Ospedale Pediatrico Bambino Gesù (1702_OPBG_2018), Rome. Written informed consent to participate in this study was provided by the participants' legal guardian/next of kin. Written informed consent was obtained from the minor(s)' legal guardian/next of kin for the publication of any potentially identifiable images or data included in this article.

## Author contributions

Conceptualization: MP and ES. Methodology and supervision: MP and MT. Investigation/experimental procedures: MN, SB, SP, and CM. Data collection and data curation: ES, MN, SB, FR, FM, MB, and AF. Writing-original draft preparation: ES, MN, and MB. Preparing figures and table: MN, SP, FR, ES, and MB. Writing-critical revision: MN, MP, and MT. All authors have read and agreed to the submitted version of the manuscript.
