# Supplementary figures and images for: Case report: Novel compound heterozygosity for pathogenic variants in MED23 in a syndromic patient with postnatal microcephaly
Source: Front Neurol. 2023 Feb 7;14:1090082. doi: 10.3389/fneur.2023.1090082 (PMC9941528; doi:10.3389/fneur.2023.1090082)

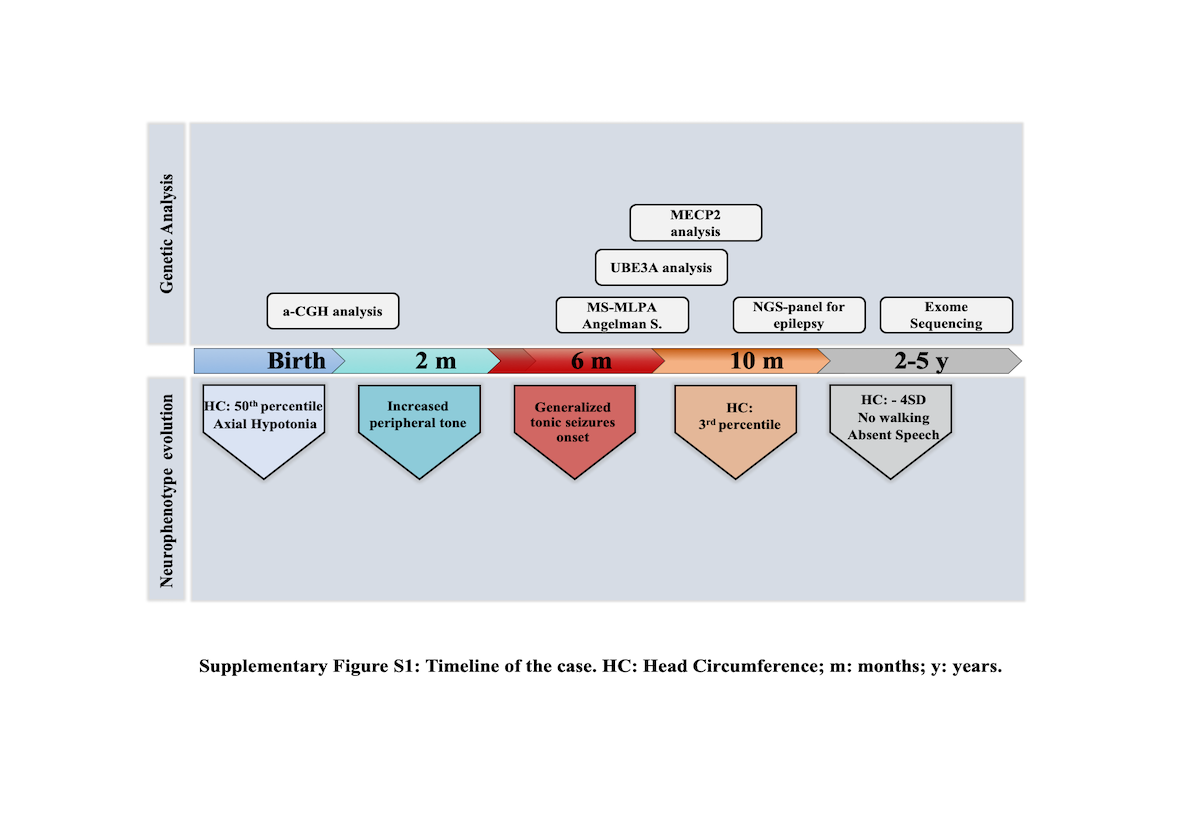

Supplement: Supplementary file 1 [file Image_1.TIFF]
