# Supplementary material for: Case report: Novel compound heterozygosity for pathogenic variants in MED23 in a syndromic patient with postnatal microcephaly
Source: Front Neurol. 2023 Feb 7;14:1090082. doi: 10.3389/fneur.2023.1090082 (PMC9941528; doi:10.3389/fneur.2023.1090082)
Supplement: Supplementary file 2 [file Table_1.DOCX]

**Supplementary Table S1.** Whole exome sequencing data output.

|  | Current Case |
| --- | --- |
| Target regions coverage, 2x^1^ | 92.0% |
| Target regions coverage, 10x^1^ | 90.6% |
| Target regions coverage, 20x^1^ | 88.0% |
| Average sequencing depth on target^1^ | 105x |
| Number of variants with predicted functional effect | 16,430 |
| Novel, clinically associated, and unknown/low frequency variants^2^ | 401 |
| Putative disease genes (Autosomal Recessive inheritance)^3^ | 2^4^ |
| Candidate genes (Autosomal Recessive inheritance) | 1, *MED23* |
| Putative disease genes (Autosomal Dominant inheritance)^3^ | 3^5^ |
| Candidate genes (Autosomal Dominant inheritance) | 0 |

**^1^**Referred to SureSelect ClinicalExome V.2 (Agilent).

**^2^**MAF <0.1% in gnomAD V. 2.0 database, and frequency <2% in our *in-house* database.

**^3^**Filtering retained genes with functionally relevant variants by excluding variants predicted as benign by CADD (scaled score <15) and M-CAP algorithms or benign/likely benign by interVar.

**^4^***MED23* (c.1831C>T, p.Arg611Trp; c.383G>A, p.Gly128Glu), *CFTR* (c.489+3A>G; c.2991G>C; p.Leu997Phe)

**^5^***LONP1* (c.1881delC, p.Val628fs), *NPIPB11* (c.1676C>G, p.Thr559Arg), *MUC19* (c.13536C>A, p.Asn4512Lys).
